# Alpha-synuclein seeding amplification assays in Lewy body dementia: a brief review

**DOI:** 10.1186/s13024-025-00868-3

**Published:** 2025-07-01

**Authors:** Maria Bregendahl, Zeynep Bengisu Kaya, Wolfgang Singer, Pamela J. McLean

**Affiliations:** 1 Center for Clinical and Translational Sciences, Mayo Clinic, 4500 San Pablo Road S, Jacksonville, FL 32224 USA; 2 Department of Neuroscience, Mayo Clinic, 4500 San Pablo Road S, Jacksonville, FL 32224 USA; 3 Mayo Clinic Graduate School of Biomedical Sciences, Mayo Clinic, 200 First St. SW, Rochester, MN 55905 USA; 4 Department of Neurology, Mayo Clinic, 200 First St. SW, Rochester, MN 55905 USA

**Keywords:** Lewy Body Dementia, Synucleinopathies, Alpha-synuclein, Biomarker, Seeding amplification assays, RT-QuIC, PMCA

## Abstract

Lewy body dementia (LBD), which includes dementia with Lewy bodies (DLB) and Parkinson’s disease dementia (PDD), is characterized by cognitive decline, sleep disturbances, motor dysfunction, and other debilitating clinical symptoms. Neuropathologically, LBD is characterized by the progressive accumulation of alpha-synuclein (aSYN) in vulnerable cellular populations in the brain. Diagnosing LBD is challenging due to the overlap of clinical symptoms with Alzheimer’s disease (AD) and other neurodegenerative disorders with current diagnostic tools, including clinical examinations by specialized neurologists and brain imaging, limited by accessibility. Taken together, LBD is often misdiagnosed, especially at early disease stages. Seed amplification assays to detect pathogenic aSYN (aSYN SAAs) are emerging as promising tools to detect aSYN pathology in biological specimens. These assays amplify trace amounts of misfolded aSYN, enabling their potential detection in brain, CSF, saliva, skin, and blood. This review compares the sensitivity and specificity of aSYN SAAs across different biological samples and explores the potential of the assay as a diagnostic in LBD. We also highlight challenges that will need to be addressed going forward if the aSYN SAA is to be widely adopted as a diagnostic test. Despite current limitations, aSYN SAAs hold promise for early and precise diagnosis, paving the way for targeted treatments that could significantly improve patient care and outcomes.

## Introduction

Lewy body dementias (LBD), which include dementia with Lewy bodies (DLB) and Parkinson’s disease (PD) dementia (PDD), rank as the second most prevalent neurodegenerative form of dementia in patients older than 65 years old [1]. In the United States, an estimated 1.4 million individuals are affected by LBD, with the incidence increasing with age [2], and more men affected than women [2]. Pathologically, LBD is characterized by the accumulation of misfolded alpha-synuclein (aSYN) protein in neurons, forming Lewy bodies (LBs) and Lewy neurites (LNs) [3, 4]. Although the full spectrum of normal physiological functions of aSYN remain indeterminate, evidence supports an important role in synaptic vesicle trafficking [5] and neurotransmitter release [6]. Adding to the complexity, LBD shares overlapping clinical symptoms with AD, and other neurodegenerative disorders complicating both diagnosis and treatment.

Currently, neuropathologic confirmation is the gold standard for diagnosing LBD, relying on the identification of Lewy pathology in specific regions of the brain [1]. This highlights the need for antemortem diagnostic tests to detect LBD before clinical symptoms fully manifest, allowing for early therapeutic intervention.

Recent advances in seed amplification assays (SAAs), such as real-time quaking induced conversion (RT-QuIC) and protein misfolding cyclic amplification (PMCA), have enabled the detection of misfolded aSYN pathogenic seeds in body fluids and tissue that appear to correlate with the presence of clinical symptoms in living individuals [7–11]. SAAs not only enable the possibility of definitive antemortem diagnosis but also provide a foundation for redefining LBD using biological markers like aSYN aggregation, rather than relying solely on clinical criteria. This shift is central to two emerging frameworks: the neuronal aSYN disease integrated staging system (NSD-ISS) proposed by Simuni et al., which defines disease stages using biological anchors of aSYN pathology and dopaminergic dysfunction [12], and the SynNuerGe proposal by Höglinger and Lang, which similarly advocates for a biology based classification system to improve diagnostic precision, guide therapy development, and better understand disease progression [13]. Both emphasize the need to move beyond traditional clinical diagnoses and instead use biomarkers—particularly SAA-based detection of aSYN—to redefine and stage Lewy body diseases from their earliest, even preclinical stages.

In this review, we discuss the current status of aSYN SAAs. We focus on comparing the different biological samples used and highlighting the differences across assay types, addressing the challenges in assay implementation and outlining future directions for assay application in advancing both the understanding and management of LBD.

### Differentiating LBD from AD

LBD and AD are distinct neurodegenerative disorders with different underlying proteinopathies. LBD is characterized by the widespread abnormal accumulation of aSYN, which forms LBs and LNs, along with the degeneration of dopamine-producing cells in the tegmentum and cholinergic cells in the basal forebrain [14]. In contrast, AD is marked by the presence of amyloid-β (Aβ) plaques and neurofibrillary tangles composed of hyperphosphorylated tau [15]. LBD primarily impacts the brainstem and corticolimbic regions [14], whereas AD affects the hippocampus and cortical regions [15]. Moreover, AD pathology often coexists in LBD, more so in DLB cases [16] than PDD [17, 18].Although LBD and AD share common clinical features, such as cognitive decline, there are important distinctions in their clinical presentation. AD typically begins with a gradual cognitive decline, often with early impairment in memory and executive dysfunction, including difficulty with planning and problem-solving [19]. In fact, a study found that memory problems were the presenting complaint in 100% of patients with AD compared to 94% of patients with DLB and 67% of patients with PDD, highlighting key differences in how memory deficits manifest across these disorders [20].

### Subtypes of LBD: DLB and PDD

LBD serves as an umbrella term for two clinical conditions: DLB and PDD, which are currently differentiated by the timing of cognitive and motor symptom onset [21, 22]. In DLB, cognitive decline occurs either before or within one year of the onset of parkinsonism, defined as the presence of motor symptoms such as tremor, rigidity, and bradykinesia. In addition to cognitive decline, DLB is characterized by fluctuations in cognition, visual hallucinations, autonomic dysfunction, and idiopathic rapid eye movement (REM) sleep behavioral disorder (iRBD) [22]. In contrast, PDD is diagnosed when changes in executive function, attention and memory develop more than a year after a well-established PD diagnosis. [21]. This arbitrary "one-year rule" provides a useful, albeit imprecise, framework for differentiating DLB and PDD.

### Current LBD diagnosis and assessment

**Fig. 1:**
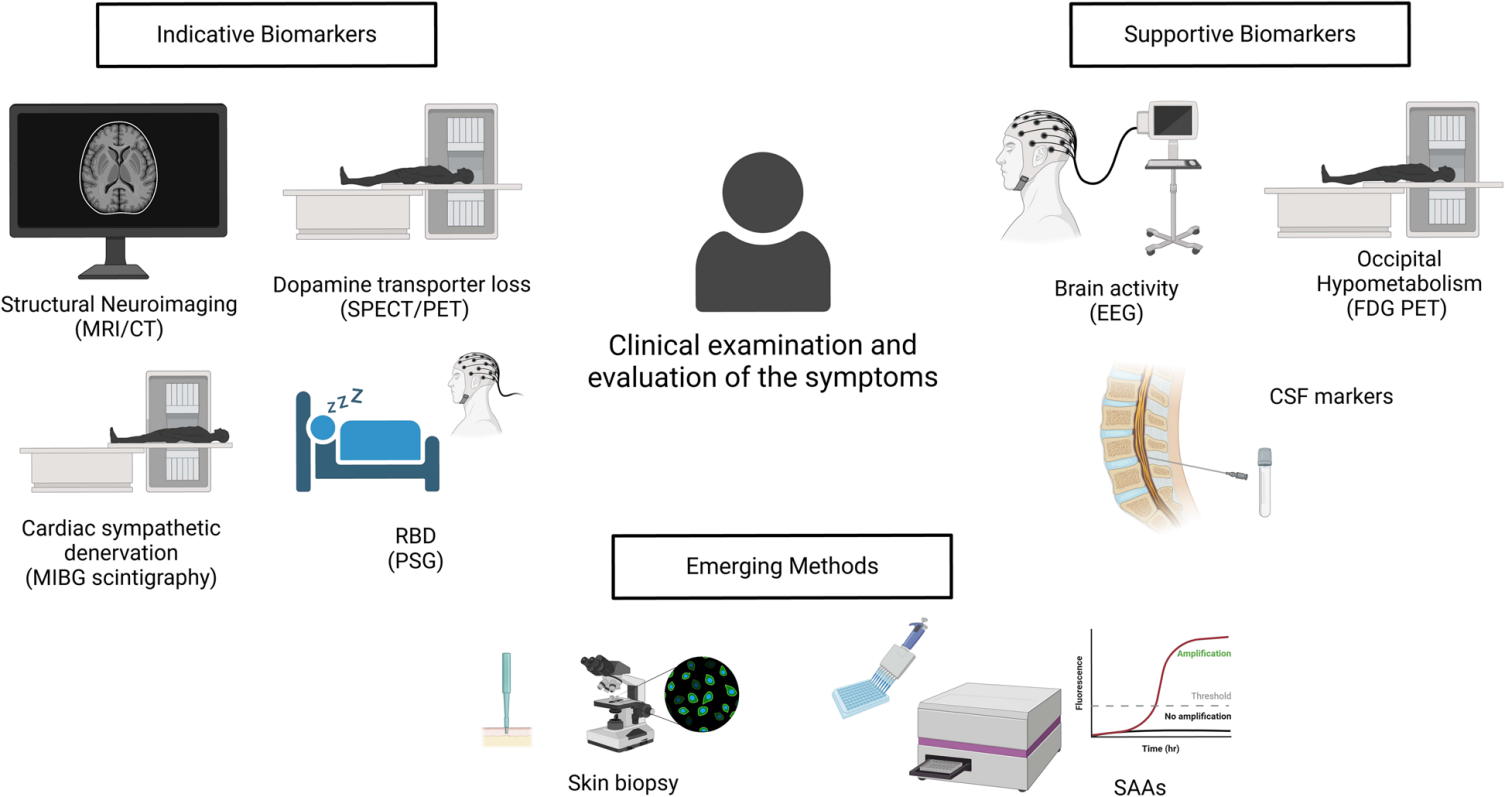
Diagnostic methods for LBD. This figure summarizes diagnostic tools used in LBD diagnosis, categorized into indicative, supportive, and emerging methods by current clinical consensus guidelines. Indicative Biomarkers and structural neuroimaging (Top Left): Tools specific to LBD that assist in diagnosis, including dopamine transporter imaging (123I-FP-CIT SPECT) for striatal dopamine loss, MIBG scintigraphy for cardiac denervation, PSG with EEG for REM sleep behavior disorder. Supportive Biomarkers (Top Right): Additional methods, such as quantitative EEG, CSF analysis to measure aSYN, amyloid-beta, tau levels, FDG-PET for occipital hypometabolism. Emerging Diagnostic Methods (Bottom): Relatively new techniques like immunohistochemistry of skin biopsies and aSYN SAAs offer potential for sensitive and early detection of LBD pathology. These biomarkers provide a comprehensive framework for improved diagnosis and monitoring of LBD. Figure was created with BioRender.com

Diagnosing LBD in clinical settings involves a comprehensive approach that integrates neurological exam, cognitive assessments, imaging studies, and often cerebrospinal fluid (CSF) collection and analysis (Fig. 1). Standard cognitive tests, such as mini-mental state examination (MMSE) or the Montreal cognitive assessment (MoCA) are frequently used in the office to evaluate cognitive function. While these assessments can help identify global cognitive impairment, formal neuropsychometric testing provides a more sophisticated and better differentiation of DLB from AD and other dementias. These tests often evaluate memory tasks, processing speed, executive function, as well as divided and alternating attention [22]. Neuroimaging with computed tomography (CT) or magnetic resonance imaging (MRI) is utilized to explore structural brain changes [23], while dopamine transporter imaging (DaTscan) helps assess striatal dopaminergic loss [24]. Diminished dopamine transporter availability in DLB and PDD, distinguishing them from AD, can be demonstrated through imaging techniques such as single-photon emission computed tomography (SPECT) or positron emission tomography (PET) [22, 25]. PET imaging using ^18^F-fluorodeoxyglucose (FDG) assesses regional glucose metabolism, often showing reductions in the occipital and parietal lobes in DLB. This contrasts with AD, where the metabolic decline primarily occurs in the temporal and parietal lobes [26].

^123^I-metaiodobenzylguanidine (MIBG) myocardial scintigraphy, quantifies reduced postganglionic sympathetic cardiac innervation in LBD patients, which is indicative of myocardial sympathetic nerve damage [27]. However, in the United States, the widespread use of MIBG myocardial scintigraphy has faced obstacles due to limited standardization, availability issues, and challenges with regulatory approval and reimbursement, limiting its clinical implementation.

EEG is considered another supportive method in DLB diagnosis, as it can show prominent posterior slow wave activity and temporal slow wave activity. However, it is not commonly used for this indication due to its low specificity and sensitivity, particularly in the earlier stages of the disease [28].

Polysomnography (PSG) is used to confirm iRBD and is recognized as a valuable tool for LBD diagnosis as iRBD may precede cognitive decline, with a prevalence of 76% among DLB cases [22]. CSF and plasma biomarkers for AD, such as Aβ40, Aβ42, and their ratio, and phosphorylated tau (pTau181, pTau217), can help differentiate LBD and AD or may identify co-pathology [29, 30].

### aSYN seed amplification assay

SAAs targeting aSYN (aSYN SAAs), are emerging as a valuable tool to detect misfolded aSYN aggregates, a hallmark of alpha-synucleinopathies. SAA works by amplifying minute amounts of misfolded aSYN, or “pathogenic seed” in biological samples, to detect the presence of seeding-competent aSYN that is capable of inducing aggregation [31, 32]. Leveraging this advancement, a significant development in the field is the availability of this assay outside of research laboratories as a commercial test in the United States [33]. Although the methodology requires further validation and homogenization and is currently still best suited for research applications or to provide complementary diagnostic information, this assay reflects the evolving landscape of neurodegenerative disease diagnosis, with the potential to enhance LBD diagnosis, paving the way for the development of comprehensive biomarker panels.

### Background of aSYN SAA

The aSYN SAA grew from research on protein misfolding and aggregation, particularly in the study of prion diseases, such as Creutzfeldt Jakob Disease (CJD). In the early 1990s, Stanley Prusiner and colleagues demonstrated that misfolded prion protein (PrP) could act as a “seed” triggering normal PrP to convert into a protease-resistant, misfolded form, PrP^Sc^ [34, 35]. This self-propagating seeding process highlighted the infectious nature of prions and provided a mechanistic framework for understanding how misfolded proteins propagate disease in neurodegenerative disorders.

### Origins of prion disease

The prion hypothesis was initially met with skepticism but gained support through innovative experiments, such as PMCA, which demonstrated the protein-only nature of prion infectivity. In the early 2000s, Dr. Claudio Soto and his team pioneered PMCA, a cell-free technique to amplify misfolded protein seeds through iterative cycles of sonication and incubation. In this process, sonication is applied to fragment fibrils, creating new seeding ends, while incubation promotes conversion of normal PrP into the misfolded form [36]. This technique enabled detection of minute amounts of PrP^Sc^ in biological samples and effectively mimicked species-and-strain specific prion propagation in vitro [37, 38]. In 2011, Atarashi et al. introduced RT-QuIC, a method similar to PMCA that enables real-time monitoring of protein aggregation. RT-QuIC uses continuous shaking to facilitate the interaction between misfolded protein seeds and recombinant monomeric proteins, inducing aggregation. This process is tracked using Thioflavin T (ThT), a dye that fluoresces upon binding to amyloid-fibrils, enabling sensitive detection of misfolded proteins [39].

A key advantage of SAAs is their adaptability in substrate composition, with variations ranging from brain-derived homogenates [36, 40] to recombinant prion proteins that can be full-length or truncated to optimize reaction efficiency [41]. Detection methods for prion SAAs typically involve either proteolytic digestion and immunoblotting [36, 42] to confirm presence of aggregated forms of prion proteins or fluorescence-based detection to monitor the kinetics of protein aggregation real-time [41]. These early protocols, initially developed for prion disease, established the foundation for adapting SAAs to other proteinopathies, such as tauopathies [43, 44] and synucleinopathies [7, 9, 31, 45], where the aggregation of tau and aSYN, respectively, play central roles in disease progression.

### aSYN seeding and aggregation

aSYN is a 140 amino acid presynaptic protein encoded by the *SNCA* gene [46]. It is highly abundant in the brain, particularly in the striatum, midbrain, and cortex [1]. Under physiological conditions, aSYN is membrane-associated, where it plays a critical role in synaptic function by regulating synaptic vesicle trafficking and neurotransmitter release through interaction with synaptic membranes and lipid rafts. In its native state, aSYN exists primarily as a monomer with an intrinsically disordered structure, facilitating these interactions. However, under pathological conditions, aSYN dissociates from synaptic membranes, undergoes conformational changes, misfolds and aggregates into fibrils characterized by intermolecular β-sheet structures, which are hallmarks of synucleinopathies [47].

The seeding-nucleation model, first described by Lansbury and colleagues [48], provides a framework for understanding the pathological aggregation of aSYN. This model suggests that the aggregation process begins with a slow, thermodynamically unfavorable step involving the formation of a nucleus—a misfolded oligomer that serves as a seed. Once formed, this nucleus catalyzes the rapid recruitment of native, soluble aSYN monomers, driving the elongation of fibrils in a highly favorable and exponential process. These fibrils can fragment, producing new seeds that further amplify aggregation. This mechanism explains how misfolded aSYN can drive the formation of fibrillar aggregates and spread abnormalities between cells and tissues [49].

Importantly, both intrinsic and extrinsic factors may influence the aSYN aggregation process, modulating seeding kinetics, structural strain properties, and pathological spread. Disease-associated mutations in *SNCA* (e.g., A53T, E46K) can alter the biophysical properties of aSYN, promoting aggregation and influencing fibril conformation [50]. Similarly, extrinsic factors such as oxidative stress, lipid membrane composition, and post-translational modifications (PTMs) including phosphorylation, ubiquitination, and truncation may impact aSYN’s aggregation and seed competence [51–55]. In a study by El-Agnaf’s group, phosphorylation at serine 129 (pSer129) was found to inhibit fibril formation, seeding activity, and cytotoxicity, rather than promoting pathology. Compared to its non-phosphorylated form, pSer129 modified aSYN exhibited lower seeding efficiency and slower aggregation kinetics [55] in the aSYN SAA. These findings suggest that certain PTMs such as pSer129 may act as inhibitory or regulatory modulators of aSYN pathology. These modulators not only contribute to heterogeneity across synucleinopathies but potentially influence the SAA output.

**Fig. 2:**
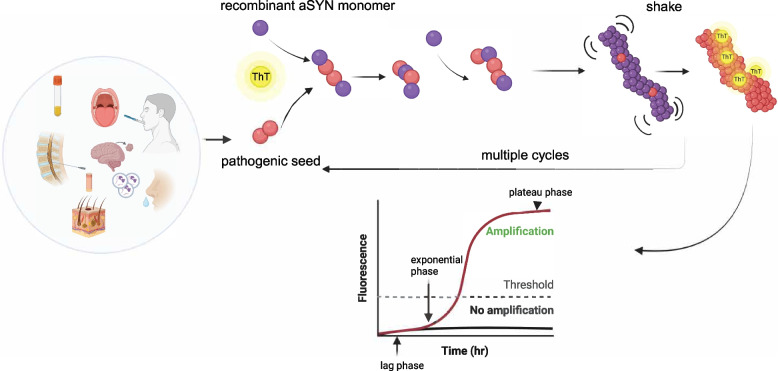
Schematic representation of αSYN SAAs. Human biological samples (e.g., cerebrospinal fluid, blood, mucosa, brain-derived extracellular vesicles from blood, skin, or brain tissue) are obtained from patients with synucleinopathies. These samples contain seed-competent α-synuclein (αSYN) aggregates capable of triggering the aggregation of monomeric αSYN added to the reaction. Through cycles of shaking and incubation, protein misfolding and aggregation are exponentially amplified. The process is monitored in real-time using the fluorescence of Thioflavin T (ThT), an amyloid-binding dye. Positive seeding activity is determined when the fluorescence signal surpasses a predefined threshold, reflecting the characteristic sigmoidal kinetics of protein aggregation (ThT fluorescence vs time), including a lag phase (initial nucleation period with no detectable increase in fluorescence), an exponential phase (rapid fibril growth and fragmentation, marked by a steep rise in fluorescence), and a plateau phase (substrate depletion and with no further increase in fluorescence). Figure was created using BioRender.com

Over time, both PMCA and RT-QuIC have been adapted to study alpha-synucleinopathies. These adaptations, now collectively referred to as aSYN SAAs, have proven effective in detecting misfolded aSYN in CSF [7, 8, 56, 57], extracellular vesicles from plasma and serum [10, 58], and tissues like skin [32, 59–61], submandibular glands [62, 63], and olfactory mucosa [64, 65]. To illustrate the principles underlying aSYN SAA, a schematic representation of the assay’s mechanism is provided below (Fig. 2). The diagram highlights the key steps involved in amplification of misfolded aSYN, displaying how the assay leverages the unique properties of protein aggregation to facilitate real-time detection.

### aSYN SAA kinetics and parameters

SAAs leverage the nucleation-polymerization model of protein aggregation, which follows a characteristic sigmoidal curve composed of three distinct phases: a lag phase, an exponential phase, and a plateau phase. These phases collectively reflect the molecular processes during the assay offering insight into the amount, conformation, and biological activity of the seeds present in the sample, as well as the broader reaction environment, including buffer composition, temperature, shaking protocol, and the presence of additives such as sodium dodecyl sulfate (SDS) and salts [7, 66–68].

The *lag phase* represents the nucleation period, during which seed-monomer interactions begin but no measurable ThT fluorescence is detected. This phase is influenced by multiple factors, including seed concentration, conformational integrity, and physicochemical compatibility of seeds and substrate. For example, Shahnawaz and colleagues demonstrated that CSF from PD patients exhibited significantly shorter lag times compared to controls [8], while Bargar et al. [69] found that DLB samples, both from brain homogenates and CSF, exhibited significantly shorter lag times than PD, suggesting that distinct populations differ in nucleation efficiency. Beyond the seeds themselves, properties of the substrate also influence early-phase kinetics. Kang et al. showed that N-terminal acetylation of aSYN modulates structural dynamics and slows aggregation [70] while Al-Azzawi et al. demonstrated that aSYN monomer, purified using an osmotic shock method (OSM), resists spontaneous aggregation and produces longer, more stable lag phases, improving reproducibility across unseeded reactions [71].

Once nucleation occurs, the *exponential phase* begins, marked by a steep rise in ThT fluorescence corresponding to fibril elongation and fragmentation. The rate of this increase quantified via slope or protein aggregation rate (PAR) offers insight into amplification dynamics. Bargar et al. [69] observed that DLB seeds produce higher PAR values than PD, and Shahnawaz et al. [8] similarly noted disease-dependent differences in aggregation rates, likely reflecting structural variation among pathological seeds. Notably, Al-Azzawi et al. found that OSM substrates, though resistant to de novo aggregation, maintained strong and dose dependent responsiveness to pathological seeds, indicating that both substrate behavior and seed characteristics shape the propagation kinetics of the assay [71].

The reaction eventually reaches the *plateau phase*, during which ThT fluorescence stabilizes as monomer is exhausted, or fibril growth reaches equilibrium. The maximum fluorescence signal (Fmax) is frequently used as a relative indicator of overall aggregation, though it should be interpreted as a direct measure of total fibril mass. However, changes in substrate type and buffer composition, including SDS levels and shaking speed, significantly affect both signal amplitude and kinetics [72]. Additionally, post-plateau drops in fluorescence can be observed, possibly reflecting fibril fragmentation or dye dissociation. These findings, along with those of Candelise et al. [72], showed that pH, polyamines, and divalent ions can influence aSYN misfolding, highlight that Fmax reflects not only aggregate quantity, but also dye accessibility, fibril morphology, and reaction microenvironment. Therefore, plateau values are most informative when contextualized alongside lag and amplification metrics within a rigorously standardized assay.

The kinetic behavior of aSYN SAA, shaped by the interplay of seed structure, substrate properties, and assay conditions, has laid the foundation for the application across a range of biological specimens. As the field has advanced, the development of aSYN SAA as a tool for detecting aSYN aggregates has been critical in understanding LBD. Initial applications of SAA detecting misfolded aSYN in brain, CSF and other tissues demonstrate the capability to differentiate between various synucleinopathies [9, 45, 73]. To further explore the role of sample types in aSYN SAA, it is important to compare the relative utility, strengths and limitations. A positive classification in SAA studies is typically based on the number of replicates exceeding a predefined fluorescence threshold within a set time frame, often accompanied by additional parameters such as lag phase or cut-offs. Table 1 summarizes key studies using brain and CSF samples, highlighting disease groups, assay type, and how these classification criteria have been operationalized across protocols.

**Table 1:** Key studies on aSYN SAA using brain and CSF samples

Sample	aSYN SAAs	Disease Groups	Fluorescence Threshold	Positive criterion based on replicates	Cut-off (hr)	Lag phase (hr)	Ref.
Brain and CSF	RT-QuIC	Brain: DLB, PD, AD, Controls; CSF: PD, DLB, at risk, Controls	Time to 5000 RFU	≥1/2	120	Brain: 50	[7]
						CSF: 60-100	
Brain and CSF	RT-QuIC	PD, DLB, AD, Controls	Mean fluorescence of first 10 cycles ± 10 SD of all samples	≥2/4	Brain: 70; CSF: 20-40	Brain: ~10; CSF: 20-40	[56]
Brain and CSF	RT-QuIC	LBD, AD, CJD, Controls	30% of median Fmax	≥3/4	60	NS	[57]
Brain	RT-QuIC	DLB, other NDs	>120 au	≥3/6	96	24-48	[66]
Brain	RT-QuIC	DLB, PD, Controls	Signal increase >50% of baseline	≥2/3	~60	<10	[74]
Brain	RT-QuIC	PD, DLB, Controls	RFU >5 SD	≥2/3	100	41-77 (TC) 14-26 (FC)	[75]
Brain	RT-QuIC	AD+LB, AD, Controls	Mean fluorescence of first 10 cycles ± 25 SD of all samples	≥2/3	60	~10-20 (AD+LB)	[76]
CSF	PMCA	PD, DLB, Controls	50 RFU	3/3	~50	10-20	[8]
CSF	PMCA	PD, DLB, MSA, Controls	RFU >5 SD	≥2/2	360	~120 (PD/DLB)	[9]
CSF	aSYN SAA	PD, iRBD/DLB*, Controls	Time to 5000 RFU	3/3	50	NS	[31]
CSF	aSYN SAA	Sporadic PD, Genetic PD, Parkinsonism with SWEDD, Prodromal, Nonmanifesting carriers, Controls	No fixed threshold: maximum fluorescence per replicate used in probabilistic algorithm to classify positive/negative	3/3	150	NS	[77]

*CSF*-cerebrospinal fluid, *RT-QuIC-* Real-Time Quaking Induced Conversion assay, *DLB-* Dementia with Lewy bodies, *PD-* Parkinson’s Disease, *ND-* neurodegenerative disorders, *AD-* Alzheimer’s Disease, *CJD-* Creutzfeldt- Jakob Disease, *RFU-* relative fluorescence unit, *SD-* standard deviation, *NC-* negative controls, *Fmax* maximum fluorescence intensity, *au*-arbitrary unit. *TC-* Temporal cortex, *FC-* Frontal cortex, *AD + LB-* (AD w/ Lewy Body pathology), *NS-* Not specified, *SWEDD-* scans without evidence of dopamine deficiency

## Biological specimens for aSYN SAA

### Brain and CSF

At present post-mortem brain tissue and its associated neuropathology remains the only method to confirm LBD with 100% certainty [66]. This makes post-mortem brain indispensable for validating the presence of pathological aSYN aggregates and understanding disease mechanisms and provides an ideal substrate for validating seeding kinetics and strain-specific properties. For instance, Poggilioni et al. reported regional differences in seeding activity, with temporal and frontal cortices exhibiting distinct aggregation profiles, which begs the question if SAA can be applied to monitor disease progression and clinical heterogeneity [75].

Post-mortem brain tissue permits the validation of assay parameters such as fluorescence threshold, lag phase, and replicates required for a positive readout. However, regional variability complicates the interpretation of results and highlights the importance of selecting representative brain tissue for assay validation. Candelise et al. highlighted strain-specific seeding kinetics between DLB and PD brain samples, expanding on these regional differences. Faster amplification rates in DLB suggested unique structural properties of aSYN aggregates, potentially reflecting differences in disease progression [74]. Manne et al. demonstrated robust performance in detecting aSYN aggregates in both DLB and PD cases, with brain tissue showing shorter lag phases (~ 10–40 h) than CSF samples from an independent cohort of PD cases [56]. This reflects the presence of more seeding-competent seeds in brain homogenates, which may be attributed to their higher pathological burden and aggregation profile. Nishida’s group further explored seeding dynamics across DLB subtypes, revealing that diffuse neocortical DLB exhibited higher sensitivity than limbic subtypes, likely due to differences in aSYN seed concentration across brain regions [78].

Jin et al. provided a unique perspective by investigating the impact of genetic factors, specifically apolipoprotein ε4 (APOE4), on aSYN seeding activity in brain tissue [76]. Their study included over 450 autopsy-confirmed AD cases with and without coexistent LB pathology. SAA successfully distinguished AD cases with LB pathology (AD + LB) from those without AD based on PAR and Fmax. Notably, the presence of APOE4 was associated with significantly higher seeding activity in AD + LB cases, suggesting that genetic risk can modulate SAA assay output and potentially impact its diagnostic interpretation [76].

Post-mortem brain tissue plays an important role in validating aSYN SAA, but its utility is largely retrospective. In contrast, CSF offers a window into ongoing disease processes in living individuals. Fairfoul et al. was the first group to utilize aSYN SAA and successfully detected aSYN aggregates in CSF with high sensitivity and specificity, reporting 92% sensitivity for DLB and 95% sensitivity for PD, both with 100% specificity. This study established the feasibility of using aSYN SAA to distinguish DLB and PD from AD, emphasizing its potential for early diagnosis [7].

The 2017 study by Shahnawaz et al. established aSYN-SAA as a highly sensitive (88.5%) and specific (96.9%) biochemical diagnostic tool for PD, capable of detecting as little as 0.1 pg/mL of aSYN oligomers and correlating aggregation kinetics with disease severity [8]. Building on this, the 2020 follow-up study [45] refined aSYN-SAA capabilities to differentiate PD from multiple system atrophy (MSA) by identifying strain-specific aggregation patterns: PD-derived aggregates exhibited higher fluorescence intensity and longer helical twists, whereas MSA aggregates formed more rapidly, plateaued at lower fluorescence, and had higher β-sheet content. Importantly, the aggregation properties of CSF-derived aSYN closely mirrored those found in brain tissue, reinforcing CSF’s value as a biomarker source [45]. The study also found that MSA-derived aggregates were more cytotoxic than those from PD, further emphasizing their structural and functional differences [45].

A similar study by Bentivenga et al. expanded on this seminal work by analyzing 269 autopsy-confirmed cases, including a subset with matched antemortem CSF samples. This unique study design allowed for direct correlation between seeding assay results and neuropathological findings, providing robust validation for the diagnostic utility of aSYN SAAs. Their analysis demonstrated 100% sensitivity for detecting LB pathology in limbic and neocortical stages but highlighted challenges in early-stage detection, such as Braak 1 and 2, where sensitivity was only 37.5%. Moreover, amygdala-predominant LBD showed intermediate sensitivity (50%), emphasizing the assay's limitations in early and localized pathology [57].

Advances in aSYN SAAs have enabled subtype differentiation. Singer and colleagues showed that aggregation kinetics in CSF could distinguish MSA from PD/DLB with 97% sensitivity and 93% specificity, using kinetic profiles alone [9]. MSA exhibited faster aggregation onset, but lower fluorescence signals compared to the slower, sustained signal observed in PD and DLB. In a follow-up study, the same group applied aSYN SAA with neurofilament light chain (NfL) to prodromal patients with pure autonomic failure. The combination of these biomarkers helped identify patients likely to convert to MSA rather than PD/DLB, reinforcing the diagnostic value of aggregation kinetics in early disease stages and suggesting prognostic potential [79].

In a large-scale cross-sectional study of the Parkinson's progression markers initiative (PPMI) cohort, Siderowf et al. evaluated CSF aSYN SAA performance in a diverse population, including patients with PD, prodromal individuals, healthy controls, and non-manifesting mutation carriers [77]. The assay demonstrated high diagnostic accuracy, with a sensitivity of 87.7% and specificity of 96.3% for distinguishing PD from controls. Notably, sensitivity reached 98.6% in sporadic PD patients with olfactory deficits but was reduced in leucine rich repeat kinase (LRRK2) mutation carriers (67.5%) and normosmic PD patients (78.3%), suggesting a molecular heterogeneity in PD subtypes. This variability aligns with the findings of Sekiya et al. [80] and Jensen et al. [81] who used proximity ligation assay to detect abundant oligomeric aSYN in LRRK2-PD cases lacking Lewy pathology. These studies allow one to speculate that the decreased SAA sensitivity in LRRK2-PD corresponds with the absence of Lewy pathology.

Additionally, in the PPMI study, aSYN SAA detected pathology in 86% of individuals with iRBD or hyposmia and 8% of asymptomatic mutation carriers, supporting its use for early detection and risk stratification. These findings further emphasize the value of aSYN SAA in tracking the molecular onset of disease and informing future biomarker-driven clinical trial designs.

#### CSF-based prodromal detection

Beyond the application in clinically diagnosed synucleinopathies, CSF-based aSYN SAAs have shown considerable promise in detecting aSYN seeding activity during prodromal disease stages. In a cross-sectional study, Iranzo et al. [82] applied aSYN SAA to CSF of individuals with polysomnography confirmed iRBD. The study found that aSYN-SAA detected misfolded aSYN in 75% of CSF samples. aSYN positive individuals also showed significantly more supportive biomarkers of prodromal PD, including hyposmia, abnormal DaT-SPECT, and orthostatic hypotension, suggesting that positivity reflects a higher pathological burden [82]. More recently, Concha-Marambio et al. [83] applied a high-throughput aSYN SAA to CSF from iRBD participants in the DeNoPA cohort and found 93.1% positivity, even in individuals sampled within one year of symptom onset. These findings provide strong evidence that misfolded aSYN can be detected consistently before the onset of overt clinical symptoms, reinforcing the potential of aSYN SAA as tools for early identification, risk stratification, and preclinical intervention in LBD.

Furthermore, recent studies have demonstrated that kinetic parameters derived from CSF-based aSYN SAAs may also provide insight into disease severity and prognosis. Brockmann et al. analyzed CSF samples from individuals with LBD and observed that faster aggregation kinetics, including shorter lag phases and higher maximum fluorescence intensities, correlated with worse cognitive performance, suggesting that SAA kinetics may reflect disease burden and progression [84]. Similarly, Bräuer et al. demonstrated that specific kinetic profiles in CSF were associated with the rate of cognitive decline in PD patients, suggesting that aSYN aggregation dynamics in biofluids can offer prognostic value [85]. These findings support the emerging utility of SAA not only for early detection but also for stratifying patients by disease trajectory and risk of cognitive impairment.

### Peripheral samples

Despite the progress in leveraging brain and CSF samples, their accessibility remains a challenge, particularly for large-scale screening and early diagnosis. Peripheral samples, such as skin biopsies, olfactory mucosa (OM), submandibular glands (SMG), and blood (serum/plasma), are gaining attention as viable sources for detecting aSYN pathology in synucleinopathies [10, 11, 58, 60–63, 86]. Their accessibility and potential for antemortem diagnostics make them attractive alternatives to brain and CSF samples. Table 2 summarizes key studies highlighting assay types and metrics, emphasizing differences in methodologies for detecting LBD (DLB, PPD, prodromal cases).

**Table 2:** Key studies on aSYN SAA using peripheral samples

Sample	aSYN SAAs	Disease Groups	Fluorescence Threshold	Positive criterion based on replicates	Cut-off (hr)	Lag phase (hr)	Ref.
Skin*^1^+ CSF	RT-QuIC	PD, LBD, Non-synucleinopathies	Skin: 45,414 au*^2^	≥2/4	60	20-50	[87]
			CSF: 54,472 au				
Skin	RT-QuIC	PD, DLB	Mean fluorescence of NC ± 10 SD	≥2/4^*3^	30	Skin: <15	[32]
						CSF: NS^*11^	
Skin	RT-QuIC	iRBD, LBD, PD	Background fluorescence signal +3 SD	≥3/4^*5^	60	37-42*^4^	[59]
Skin	RT-QuIC + PMCA	PD, LBD, MSA, AD, HC	RT: QuIC – mean value of all NC at 60 hrs. + 3 SD	≥2/4^*6^	60	10-25	[61]
OM + CSF	RT-QuIC	PD, probable DLB, prodromal DLB, DLB, AD+ DLB, other NDs, HCs	Mean fluorescence of all samples for 10 hrs. OM: ±3 SD CSF: ±10 SD	≥2/4	80	OM: 30 CSF: 25	[64]
OM	RT-QuIC	iRBD, PD	±3 SD above baseline or 15-17 hrs.	≥2/4^*7^	80	NS	[65]
SMG	RT-QuIC	PD, iLBD	Mean of first 10 cycles of all samples ±10 SD	≥2/4	Frozen tissue: 24	20 (iLBD)	[62]
					FFPE tissue^*8^:60		
Saliva	RT-QuIC	PD, MSA, controls	Max fluorescence at ≥22.72%	≥2/4	35	~2.2 (PD)	[63]
Serum (NE)	ThT-based	PD, controls	NS	=1/1	Until the ThT signal plateaued	~20 (PD)	[10]
Serum (NE)	ThT-based	Prodromal PD, PD, iRBD, HC	Mean ThT signal of HC in first 10 hrs. ±5 SD	=1/1	Until the ThT signal plateaued	Variable	[11]
Plasma (NE^*10^)	ThT-based	PD, HC	Mean ThT signal of HC in first 10 hrs. ±5 SD	=2/2	NS	5.2-12.1	[58]
Serum	RT-QuIC (IP)	DLB, MSA, PD, RBD, Parkin-PD	NS	≥2/3^*9^	120	~30	[88]

*1: different skin sites used leg, thigh, cervical, *CSF*-cerebrospinal fluid, *NC*-negative control, *au*-arbitrary unit, *RFU*- relative fluorescence unit, *2: based on the mean alpha synuclein seeding activity of control + 3 standard deviation, *3: set as 15% of the maximum value reached by any of the positive control replicates during the 30-hour run, *iRBD*- rapid eye movement sleep disorder, *4: this value is for iRBD /prodromal LBD, *5: positive sample if at least ¾ reached 6000 RFU, *HC*-non-neurodegenerative controls, *7: lag phase should be less than 50 hrs to be considered a positive sample, *7: if 2/4 they cross 100,000 RFU, *iLBD*-Lewy but not Parkinson’s Disease (*PD*), *MSA*-Multiple Systems Atrophy, *8: *FFPE* formalin-fixed paraffin embedded, *9: if sample reaches 260,000 RFU  at 120 hrs, *OM*-olfactory mucosa, *SMG*-submandibular gland, *10: Neuronal EVs, *11: *NS*-Not specified, *ThT*-Thioflavin T

#### Skin biopsies

Immunohistochemical detection of phosphorylated aSYN in skin biopsies has emerged as a promising diagnostic tool for synucleinopathies, with studies demonstrating high sensitivity and specificity for detecting aSYN in peripheral nerves [89, 90]. While these studies underscore the clinical utility of skin based aSYN detection, SAAs offer an alternative approach with enhanced sensitivity for detecting misfolded aSYN. Skin biopsies offer a minimally invasive method to detect aSYN pathology within autonomic nerve fibers. Wang et al. demonstrated that aSYN SAA applied to abdominal skin biopsies achieved 94% sensitivity and 98% specificity for detecting PD, making it a highly accurate diagnostic tool [61]. Similarly, Donadio et al. reported comparable diagnostic performance between skin and CSF assays, suggesting skin biopsies as a practical alternative for patients unwilling or unable to undergo lumbar punctures [87]. Kuzkina et al. provided additional insights by analyzing seeding activity in patients with iRBD. Their study found higher seeding activity in iRBD compared to PD, underscoring the potential of skin biopsies for early detection of prodromal synucleinopathies. SAA detected aSYN aggregation in 97.4% of iRBD patients and 87.2% of PD patients, compared to just 13% of controls, demonstrating high sensitivity. However, this was accompanied by lower specificity relative to immunohistochemistry, which showed 65.7% positivity in iRBD, 40.7% in PD, and zero in controls [60]. Nevertheless, longer lag phases observed in skin assays (~ 37–42 h) compared to brain and CSF samples highlight the need for optimized protocols to enhance sensitivity and reduce assay time [60].

#### Olfactory Mucosa (OM)

The olfactory mucosa, accessible via nasal swabbing, presents another compelling option for peripheral diagnostics. Stefani and colleagues demonstrated that aSYN SAA detected seeds in 44.4% of iRBD patients and 46.3% in PD patients, with only 10.2% positivity in controls. This yielded a sensitivity of 45.2% and specificity of 89.8%, supporting its utility for screening in early stage synucleinopathies despite moderate sensitivity [65]. Perra et al. extended these findings in a DLB cohort, showing that aSYN SAA of OM alone achieved 81.4% sensitivity and 92.1% specificity for DLB diagnosis. Notably, combining OM with CSF raised diagnostic accuracy to 100%, highlighting a synergistic effect between central and peripheral sources for biomarker confirmation. While OM SAA is promising, challenges such as sampling variability and lower sensitivity in prodromal stages underscore the need for optimized protocols and combined tissue strategies for robust early detection [64].

#### Submandibular Gland (SMG)

SMG are particularly relevant because they are involved in the production of saliva and may reflect systemic changes associated with neurodegenerative diseases. Manne et al. applied aSYN SAA to submandibular gland samples and achieved 100% sensitivity and 94% specificity for detecting PD and incidental Lewy body disease (iLBD). Notably, this assay identified seeding activity in iLBD cases that were undetectable by immunohistochemistry, demonstrating the high sensitivity of aSYN SAAs [62].

Expanding on the utility of salivary glands, Luan et al. evaluated the performance of salivary aSYN SAA in living patients with PD and MSA. Their assay distinguished PD from controls with 76% sensitivity and 94.4% specificity and showed 61.1% sensitivity for MSA. While Fmax did not differ significantly between PD and MSA, the lag phase was significantly shorter in PD, suggesting a kinetic signature that may help discriminate synucleinopathy subtypes [63].

#### Blood

Blood-based samples, including plasma, serum, and more recently extracellular vesicles (EVs) of neuronal origin, offer minimally invasive options for detecting aSYN pathology [10, 11, 88]. While aSYN is normally abundant in erythrocytes [91], the use of blood for SAA is complicated by plasma proteins that interfere with assay sensitivity and specificity [92]. Traditional SAA has struggled with these inhibitors, which can lead to false negatives or reduced detection rates.

Innovative approaches have sought to overcome these barriers. Christenson et al. showed that a blood-based, nanoparticle-enhanced aSYN SAA (Nano-QuIC) could overcome common assay inhibitors in blood and significantly improve detection sensitivity. Their method achieved 100-fold greater sensitivity than conventional RT-QuIC and successfully detected aSYN seeding activity in plasma from patients with PD [93].

Okuzumi et al. developed an immunoprecipitation(IP)-enhanced aSYN SAA assay for serum, achieving 94.6% sensitivity and 92.1% specificity for PD, and 96.4% sensitivity and 92.2% specificity for DLB, while maintaining the ability to distinguish disease-specific aSYN strains [88].

Kluge and colleagues advanced this further by isolating neuron-derived EVs (NEs) from blood and demonstrating that these harbor seed-competent aSYN. Using a modified ThT based SAA performed in single wells per sample, they reported 100% sensitivity and 100% specificity in distinguishing PD patients (*n* = 30) from controls (*n* = 50) based on seeding profiles [10]. Notably, their longitudinal analysis showed that aSYN seeds could be detected 1 to 10 years before clinical PD diagnosis, highlighting the potential of this assay for prodromal detection [11]. However, the lack of technical replicates in the assay may limit the reproducibility and generalizability of these findings.

Similarly, Schaeffer et al. applied SAA to plasma neuronal EVs and reported 98.8% sensitivity for PD detection, further confirming that enriched EVs can harbor pathology-relevant aSYN conformers [58].

While these findings are promising for PD, the application of blood-based EV assays to LBD is still limited and requires broader validation across synucleinopathy subtypes. Moreover, optimization of neuronal EV enrichment methods remains essential to minimize contamination from non-neuronal sources, which can impact assay performance.

Collectively, these findings underscore the diagnostic potential of blood-derived SAAs and call for further research to refine protocols and evaluate specificity for LBD. Standardization and cross-cohort validation will be key to translating these advances into reliable clinical tools.

## SAAs and therapeutics

Recent research has employed SAA to investigate the effects of specific drug candidates on aSYN aggregation. For example, doxycycline has been shown to exert significant effects on aSYN aggregation. Dominguez-Meijide et al. reported that doxycycline not only inhibits aSYN-associated pathologies in vitro but is also neuroprotective in an animal model. The mechanism of action for doxycycline appears to involve direct binding to aSYN aggregates which may disrupt formation and propagation. This is particularly relevant given the prion-like behavior of aSYN aggregates, which can spread through neural networks, exacerbating neurodegenerative processes [94].

An additional study by Jin et al. demonstrated that certain parkinsonian drugs such as entacapone, carbidopa, and tolcapone can alter conformation of aSYN aggregates, potentially hindering further aggregation by disrupting the interactions within aggregates. The computational models generated in this study illustrate how these drugs bind to specific residues of aSYN, suggesting distinct mechanisms of action that could mitigate mitochondrial dysfunction associated with aSYN accumulation. This highlights the potential of using SAA not only to understand the aggregation process but also to evaluate therapeutic interventions [95].

In addition to these compounds, hydrazones may also serve as effective aggregation inhibitors, particularly through their interactions with metal ions that are known to influence aSYN aggregation dynamics [96]. The ongoing exploration of hydrazones underscores the ongoing efforts to identify and develop therapeutic interventions targeting aSYN aggregation, leveraging the capabilities of aSYN SAAs to evaluate their efficacy [95].

The ability to detect trace amounts of pathological proteins in peripheral tissues, such as blood, skin and saliva, opens new avenues for less invasive and non-invasive diagnostics [63, 97]. This is particularly relevant in the context of pre-symptomatic diagnosis, where early intervention could significantly alter disease trajectories [98, 99]. The ongoing refinement of these assays, including the optimization of reaction conditions and the exploration of different biospecimens, will continue to enhance their clinical utility [100, 101].

## Challenges of aSYN SAAs

A major limitation in the current application of aSYN SAAs is the lack of standardized protocols and the inherent variability across biospecimen types. Differences in sample accessibility, composition and pre-analytical handling significantly impact assay sensitivity, specificity and reproducibility. For instance, post-mortem brain tissue, while invaluable for validating pathological aSYN aggregates and understanding disease mechanisms, is limited to retrospective analysis, and offers only an endpoint snapshot. CSF offers a more accessible alternative for living individuals, but studies have shown that sensitivity drops significantly in early stages of disease, such as Braak 1 and 2, highlighting the difficulty in capturing low concentrations of aggregates [57]. Importantly, pre-analytical variables such as blood contamination, delayed freezing and addition of detergents have been shown to significantly affect SAA performance. Even low levels of blood contamination (≥ 0.001%) and delayed freezing of CSF at 4 °C can prolong lag time and reduce fluorescence intensity, impairing sensitivity and reproducibility [102]. These findings underscore the importance of implementing rigorous, harmonized pre-analytical protocols for sample processing.

In addition to sample variability and pre-analytical handling, the quality and characteristics of the recombinant aSYN monomer used as substrate in SAAs represents a major source of assay variability. Differences in expression systems, purification protocols, aggregation state, and residual contaminants, such as endotoxin or affinity tags, can influence the monomer’s responsiveness to pathological seeds [98, 102, 103]. Notably, batch to batch differences can significantly impact seeding kinetics, while lag phase was relatively stable across batches, maximum fluorescence and area under the fluorescence-time curve (AUC) varied substantially, underscoring the need for substrate standardization to ensure reproducibility [102]. Despite ongoing efforts to generate high-quality, standardized recombinant aSYN monomer, substrate-related variability remains a key limitation to cross-laboratory consistency. Greater transparency in substrate preparation methods and development of validated reference materials may help mitigate these issues.

Peripheral biospecimens such as skin, OM, saliva, and blood offer non-invasive access to aSYN pathology and hold promise for early detection and longitudinal monitoring. However, their diagnostic utility remains limited by lower seed concentrations and matrix-specific challenges. For instance, interfering proteins, variable aggregate structures, and inconsistent assay performance across cohorts [32, 63, 72]. While promising results have been reported in prodromal conditions like iRBD [83], and in salivary samples [63], assay sensitivity remains modest compared to brain or CSF, underscoring the need for further protocol refinement and validation in early-stages of the disease.

Beyond biospecimen variability, heterogeneity in assay parameters further complicates interpretation across studies. Protocols differ widely in fluorescence thresholds, and substrate preparation, reaction conditions, and lag phase definitions. Candelise et al. notably highlighted strain-specific differences in aSYN seeding kinetics between DLB and PD demonstrating how aggregate properties can influence assay performance and lead to inconsistent results [74]. While brain tissue often exhibits shorter lag phases due to higher aggregate concentrations, peripheral samples require extended amplification times [74]. This underscores the need for standardized protocols that account for sample-specific differences to ensure consistency and comparability across studies.

Although innovative techniques like IP-enhanced aSYN SAA, as demonstrated by Okuzumi et al. [88], have achieved promising results, further refinement is needed to mitigate interference and improve robustness. Neuronal EVs offer another promising avenue, with studies by Kluge et al. and Schaeffer et al. showing their potential for capturing CNS-specific pathology in PD [10, 58]. However, the application of EV-based assays to LBD remains underexplored, and standardizing isolation and assay protocols will be critical for their broader use.

Even though certain clinical scenarios of early disease stages, namely purely autonomic failure, have shown remarkably high-sensitivity for aSYN SAAs to not only detect, but differentiate the underlying pathology, early-stage detection remains a major challenge [9]. Reduced sensitivity in preclinical and prodromal cases limits their utility for early diagnosis and intervention. Studies like those by Kuzkina et al., which focused on skin biopsies, further highlight the variability in sensitivity and the need for protocol optimization to enhance early detection capabilities [60]. Additionally, the structural and biochemical differences in aSYN aggregates not just CSF but across sample types, impact assay performance by influencing seeding kinetics and aggregation dynamics. This variability complicates interpretation and emphasizes the need for more comprehensive structural characterization.

## Future directions

To address these challenges, future efforts should prioritize standardizing assay protocols, including fluorescence thresholds, substrate selection, and lag phase definitions, to improve reproducibility and diagnostic accuracy. Enhancing assay sensitivity for peripheral samples, such as neuronal EVs and skin biopsies, will also be essential for expanding their clinical utility.

In parallel, future research should focus on the structural characterization of aSYN aggregates across various samples beyond just CSF and disease stages. Understanding these differences could aid in patient stratification, enabling targeted and personalized therapies. Longitudinal studies are also needed to validate the utility of aSYN SAAs for tracking disease progression and assessing therapeutic interventions. Furthermore, leveraging these assays for therapeutic evaluation, as demonstrated by studies investigating drugs like entacapone and doxycycline, represents an exciting avenue for advancing personalized medicine.

By addressing these challenges through targeted research and innovation, aSYN SAAs have the potential to transform the early detection and management of LBD. Improving sensitivity, standardizing methodologies, and expanding their application to minimally invasive samples will pave the way for their integration into routine clinical practice, ultimately enhancing patient outcomes and supporting the development of disease-modifying therapies.

## Conclusion

The field of aSYN SAAs have made remarkable progress in the detection and differentiation of synucleinopathies. These assays provide unparalleled sensitivity and specificity in detecting aSYN aggregates, enabling their application even in prodromal stages of disease. The exploration of these diverse sample types has not only increased accessibility but also uncovered valuable insights into the heterogeneity of aSYN aαcross different tissues.

Structural and biochemical differences in aggregates, as observed in samples like EVs or skin biopsies, could inform disease progression and stratification. Future research should focus on standardizing protocols and validating these methods across large, neuropathologically confirmed cohorts to ensure diagnostic accuracy and consistency.

By detecting and characterizing aα strains from different sample types, these assays can support patient stratification in clinical trials, ensuring targeted and effective therapeutic interventions. Moreover, the ability to monitor aSYN aggregate dynamics over time provides a powerful tool for evaluating the efficacy of disease-modifying treatments. This is particularly relevant as therapies aiming to reduce or halt aggregate propagation are developed, offering the possibility of altering disease trajectories.

## Data Availability

Data sharing is not applicable to this article as no datasets were generated or analyzed during the current study.

## Abbreviations

Aβ: Amyloid-beta
AD: Alzheimer’s disease
APOE4: Apolipoprotein ε4
aSYN: Alpha-synuclein
CSF: Cerebrospinal fluid
CT scan: Computed tomography scan
DaT: Dopamine transporter
DLB: Dementia with Lewy bodies
EEG: Electroencephalogram
EV: Extracellular vesicles
Fmax: Maximum fluorescence threshold
FDG-PET: 18F-Fluorodeoxyglucose positron emission tomography
iLBD: Incidental Lewy body disease
IP: Immunoprecipitation
iRBD: Idiopathic rapid eye movement (REM) sleep behavior disorder
LB: Lewy bodies
LBD: Lewy body dementia
LN: Lewy neurites
MIBG: 123I-metaiodobenzylguanidine
MRI: Magnetic resonance imaging
MSA: Multiple system atrophy
OM: Olfactory mucosa
OSM: Osmotic shock method
PAR: Protein aggregation rate
PD: Parkinson’s disease
PDD: Parkinson’s disease dementia
PET: Positron emission tomography
PMCA: Protein misfolding amplification assays
PrP: Prion protein
pSer129: Phosphorylated at serine 129
PSG: Polysomnography
PTMs: Post-translational modifications
REM: Rapid eye movement
RT-QuIC: Real-time quaking-induced conversion
SAAs: Seed amplification assays
SDS: Sodium dodecyl sulfate
SMG: Submandibular glands
SPECT: Single-photon emission computed tomography
